# Zhidai Decoction Inhibits Cervical Cancer through Regulation of Vaginal Microbiota

**DOI:** 10.1155/2020/8940582

**Published:** 2020-08-10

**Authors:** Suying Liu, Hua Yan, Yi Liu, Yongjun Tang, Yu Lu, Huizhi Shao, Yutong Cui

**Affiliations:** ^1^Hospital of Chengdu University of Traditional Chinese Medicine, Chengdu 610072, China; ^2^School of Basic Medical Sciences, Chengdu University of Traditional Chinese Medicine, Chengdu 611137, China; ^3^School of Public Health, Chengdu University of Traditional Chinese Medicine, Chengdu 611137, China

## Abstract

Cervical cancer is one of the top lethal malignancies among women worldwide. The current treatment methods have so many drawbacks that new treatment methods need to be developed. Zhidai Decoction (ZDD) is an effective traditional Chinese herbal formulation for gynecological diseases. Its main effect is controlling abnormal leucorrhea which is a typical early clinical manifestation of cervical cancer. However, how ZDD directly affects cervical cancer has not been addressed. In this study, we established a mouse cervical cancer U14 cell subcutaneous transplantation tumor model and took an early intervention with ZDD to evaluate the antitumor effect of ZDD. In addition, we also investigated the regulatory effects of ZDD on the vaginal microbiota using 16S rRNA analysis in this study. Our results showed that ZDD can significantly improve systemic symptoms and reduce vaginal secretions of tumor-bearing mice. Compared with the CCM group (the cervical cancer model group), in the ZDD-treated group, the tumor inhibitory rate was 37.90%, the average daily food intake of mice was increased to 5.27 ± 0.74 g (*P* < 0.05), and the survival time was obviously prolonged to 21 days (*P* < 0.05). Analysis of the sequencing results of 16S rRNA showed that the main microbial genera of the CCM group were *Pasteurella* (27.20%) and *Helicobacter* (18.50%), while those in the ZDD group were *Staphylococcus* (13.22%) and *Lactobacillus* (4.68%). It revealed that ZDD has the effect of regulating the vaginal microbiota of cervical cancer, especially in increasing the relative abundance of *Lactobacillus* and *Staphylococcus* and decreasing the relative abundance of *Pasteurella* and *Helicobacter*. The analysis also showed that ZDD could adjust microbiota structure, species abundance, and community compositions of vaginal microbiota. In conclusion, ZDD displayed inhibitory effect on cervical cancer, and it might be based on restoring the balance of vaginal microbiota. Furthermore, our conclusion supports the promotion of ZDD in the early treatment of cervical cancer.

## 1. Introduction

With an estimated 570,000 cases and 311,000 deaths in 2018 worldwide, cervical cancer is still one of the diseases that seriously endanger women's health [1]. However, the existing methods for cervical cancer treatment have obvious drawbacks, such as chemotherapy sequelae and cytotoxicity [2]. Therefore, it is necessary to explore more effective and less toxic anticancer drugs [3]. Traditional Chinese herbal formulation can act on the multiple targets through their multiple components and play an integral role in the key biological process of disease development [4] and has been widely applied in cancer therapy [5], particularly for its substantially curative effects and few side effects [6–8]. As an established formula, ZDD has originally appeared 135 years ago in Qing Dynasty. ZDD is mainly used in female genital tract diseases such as chronic cervicitis and against human papillomavirus (HPV) infections [9–12]. Some herbs in ZDD have demonstrated antitumor effects [13–15] and inhibition of the growth of bacteria [16–18]. For instance, the tuckahoe polysaccharide can directly inhibit the tumor cells and enhance the immunity activities [13]. However, the affects and the mechanism of ZDD on cervical cancer have not been addressed. On the other hand, accumulated studies displayed that HPV infection is the primary cause of cervical cancer [1], especially the persistent infection by high-risk HPV [10, 19, 20]. The persistent infection of HPV is associated with a high diversity of vaginal microbiota [21] and vaginal microbiota imbalance [22]. Therefore, the changes in vaginal microbiota may play an important role in the occurrence and development of cervical cancer [22, 23]. Hence, we hypothesize in the present work that ZDD may inhibit the process of cervical cancer through the approach of restoring the balance of vaginal microbiota. This work may provide novel pharmaceutical evidence of ZDD on cervical cancer therapy.

## 2. Materials and Methods

### 2.1. Animals and Reagents

Thirty-three female Kunming mice, with an age 6–8weeks and average weight 20 ± 2 g (Certificate: 51203500004648), were obtained from Chengdu Dashuo Experimental Animal Co., Ltd., and raised in standard conditions. All procedures of our experiments were carried out in accordance with the Guidelines for the Care and Use of Laboratory Animals and approved by the Animal Ethics Committee of Chengdu University of Traditional Chinese Medicine. U14 cervical ascites tumor cells were purchased from Beijing Silver Amethyst Biomedical Technology Co., Ltd. ZDD consists of 14 mg *Polyporus umbellatus*, 14 mg *Poria cocos*, 18 mg *Psyllium*, 14 mg *Alismatis Rhizoma*, 9 mg *Herba Artemisiae Scopariae*, 14 mg *Paeoniae Radix Rubra*, 14 mg *Moutan Cortex*, 9 mg *Cortex Phellodendri*, 9 mg *Fructus Gardeniae*, and 14 mg *Cyathula officinalis Kuan*. All crude herbs in ZDD were purchased from Sichuan Derentang Pharmaceutical Chain Co., Ltd., and prepared the aqueous extract.

### 2.2. Cervical Cancer Model-Made and ZDD Treatment

All mice were kept under standard environmental conditions and had free access to food and water for 7 d. According to the randomized block design, 33 mice were divided into three groups: the normal control group (CON) (*n* = 12), the cervical cancer model group (CCM) (*n* = 12), and the ZDD-treated group (ZDD) (7 g/kg/day) (*n* = 9). U14 cervical cancer cells were injected into the abdominal cavity of the first-generation mice; after passing three generations, U14 cell suspension was obtained (the proportion of living tumor cells >95%, the concentration of tumor cells in ascites solution was adjusted to 1∗10^8^/ml). Then, the suspension was 1:5 diluted with normal saline to obtain the final suspension (the concentration is 2∗10^7^/ml). Each mouse in the CCM and the ZDD groups was subcutaneously injected with 0.2 mL of cell suspension on the upper part of the right hind limb. All of the above operations are performed in a sterile laboratory.

The mice were intragastrically administered once a day from the first day of model-made treatment, last for 21 days. Each mouse of the CON and CCM group was administered with 0.2 mL of 0.9% normal saline solution. Mice of the ZDD group were administered with crude drug 7 g/kg, and the concentration of the solution is 0.7 g/mL. The gastric perfusion amount of all mice was 10 ml/kg per day. General conditions (activity, appetite, and mental state), tumor size, and death situation of each group were recorded daily. And the sample collection was carried out on the 22nd day of modeling. First, the cervical sampler was used to collect the vaginal microbiota for 16S rRNA gene sequencing. Then, the mice were killed and isolated, and tumor tissues were weighed to calculate the tumor inhibitory rate (TIR%). The tumor inhibitory rate (TIR%) was calculated as follows: TIR% = (1 − Wt/Wn) × 100%, where Wn is the average tumor weight of the CCM group and Wt is the average tumor weight of the ZDD group.

### 2.3. Vaginal Microbiota Analysis by 16S rRNA Gene Sequencing

Total genome DNA of vaginal microbiota samples was extracted using CTAB/SDS [24,25] method. The V4 region of 16S rRNA was amplified with the forward primer 515F (5′-GTGCCAGCMGCCGCGGTAA-3′) and the reverse primer 806R (5′-GGACTACHVGGGTWTCTAAT-3′). The amplified products were sequenced on the Ion S5^TM^ XL sequencing platform in Novogene (Beijing, China). Qualities' filtering on the raw reads was performed under specific filtering conditions to obtain the high-quality clean reads according to Cutadapt (V1.9.1, http://cutadapt.readthedocs.io/en/stable/) quality control. Sequences' analyses were performed by Uparse software (V7.0.1001, http://drive5.com/uparse/). Sequences with ≥97% similarity were assigned to the same Operational Taxonomic Units (OTUs). For species annotation, the Silva database (https://www.arb-silva.de/) was used based on Mothur algorithm to annotate taxonomic information. Beta diversity index on both weighted and unweighted UniFrac was calculated by QIIME software (Version 1.9.1). Unweighted Pair-Group Method with Arithmetic Means (UPGMA) Clustering was performed as a type of hierarchical clustering method to interpret the distance matrix using average linkage and was conducted by QIIME software (Version 1.9.1).

### 2.4. Statistical Analysis

Statistical analyses were performed using SPSS 22.0 (IBM Software, New York, USA) and drawn by GraphPad Prism version 8 (GraphPad Software Inc., 2019, San Diego, California, USA). Measurement data of the food intake were expressed as the mean ± standard deviation (SD) and were analyzed by repeated measurement variance analysis. Measurement data of the survival time were expressed as the *M* ± *Q* (*M* = median, *Q* = interquartile range) and were analyzed by Kruskal–Wallis Test. The tumor weight was expressed as the mean ± standard deviation (SD) and was analyzed by one-way ANOVA. *P* values < 0.05 were considered statistically significant.

## 3. Results

### 3.1. The Effects of ZDD on Tumor Growth

Taking the average tumor weight of the CCM group as a control, the tumor inhibitory rate (TIR %) of the ZDD group was 37.90% (>30%) (Table 1), which means it had a significant antitumor effect. The survival time of the tumor-bearing mice in the CCM group was 19.50 days, while the survival time of the mice in the ZDD group was 21 days. Compared with the two groups, the survival time of tumor-bearing mice in the ZDD group was significantly prolonged (*P* < 0.05) (Table 1). The results showed that ZDD may have a certain inhibitory effect on the growth of cervical cancer.

### 3.2. The Effects of ZDD on General Condition and Food Intake

**Figure 1:**
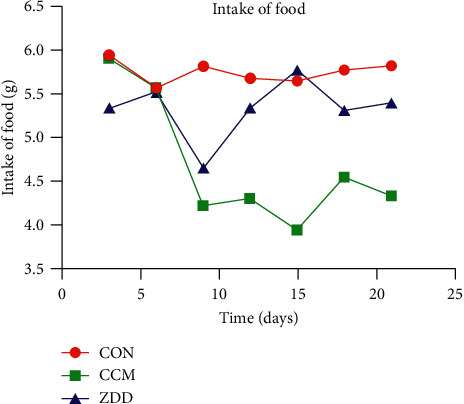
Conditions of food intake.

The mice in the CON group did not show any abnormal symptoms. They have normal intake, shiny pelage and normal fecal morphology, good mental state, and quick movements. But the tumor-bearing mice in the CCM and the ZDD groups, with the growth of the tumor, the pelage lost luster, the shapes of feces were changed, and mental state was getting worse. However, the conditions of the ZDD group were better than the CCM group, especially in the aspect of mental states and the activity. In addition, the mice of the CCM group also secreted unequal amounts of vaginal secretions, but this symptom was not observed in the ZDD the CON groups. The food intake of the tumor-bearing mice decreased gradually with the growth of the tumor. Compared with the CON group, the food intake of the CCM group was significantly decreased (*P* < 0.05), while the ZDD group was not decreased obviously (*P* > 0.05) (Figure 1 and Table 1).

### 3.3. The Effects of ZDD on Vaginal Microbiota

#### 3.3.1. Correlations between Three Groups of Vaginal Microbiota

**Figure 2:**
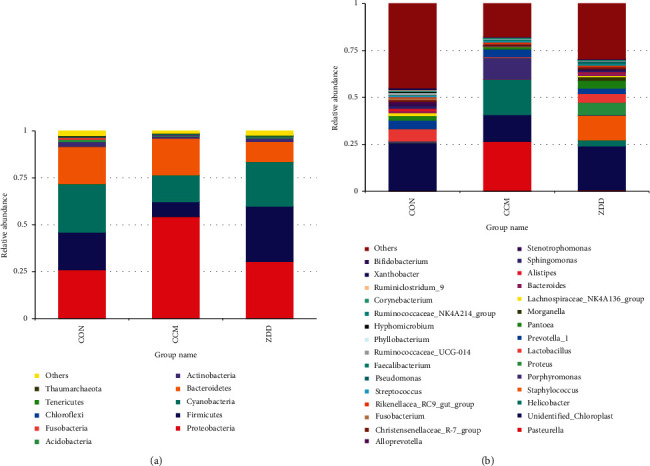
Relative abundance histogram of each group. (a) Relative abundance histogram of species at the phylum level. (b) Relative abundance histogram of species at the genus level. The different colors in the figure represent the species and relative abundance of the main dominant vaginal microbiota in the CON, CCM, and ZDD groups, respectively.

At the phylum level, Firmicutes were more enriched in the CON group (20.10%) and the ZDD group (29.19%), while in the CCM group, they were significantly reduced (7.72%). Inversely, Proteobacteria were more enriched in the CCM group (54.70%), while in the CON group (25.84%) and the ZDD group (30.41%), they were significantly reduced (Figure 2(a)). At the genus level, the most important dominant taxon in the CON group was *Lactobacillus* (6.68%). And the dominant taxa in the ZDD group were *Staphylococcus* (13.22%), *Proteus* (6.74%), and *Lactobacillus* (4.68%), while the dominant taxa in the CCM group were *Pasteurella* (27.20%) and *Helicobacter* (18.49%). Moreover, the relative abundance of the *Lactobacillus* reduced to 0.49% (Figure 2(b)). These results revealed that the relative abundance of the dominant taxa of the CON and the ZDD groups was similar, while that of the CCM group was much more different. In comparison with the CON group, the relative abundance of *Lactobacillus* in the CCM group was significantly lower by as much as 13 times, while the relative abundance of *Pasteurella* and *Helicobacter* was significantly increased. Meanwhile, the relative abundance of *Staphylococcus* in the ZDD group was much higher than that in the CCM and the CON groups (Figure 2(b)). At the species level, the results showed that *Helicobacter bilis* is the dominant species of the *Helicobacter* genus in the CCM group and *Staphylococcus lentus* is the dominant species of the *Staphylococcus* genus in the ZDD group (Table 2).

#### 3.3.2. Beta Diversity Index Analysis of Vaginal Microbiota in Each Group

**Figure 3:**
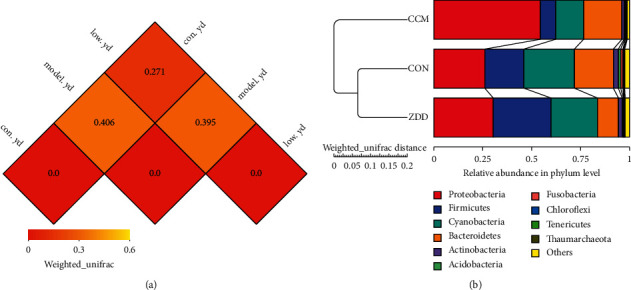
Beta diversity index analysis. (a) Beta diversity index heat map (CON = con.yd, CCM = model.yd, and ZDD = low.yd). (b) UPGMA clustering dendrograms and relative abundance histogram.

The beta diversity index uses the weighted UniFrac distance to measure the difference coefficient between the two samples. The smaller value means the smaller difference in species diversity between the two samples. In other words, there is more similar species structure and species abundance between the two groups. The CON group and the ZDD group have the lowest difference coefficient of 0.271, indicating that the two groups are the closest in terms of species structure and species relative abundance. But the difference coefficient between the CON group and the CCM group is 0.406, indicating that the two groups are diverse in terms of microbiota structure and species abundance (Figure 3(a)).

#### 3.3.3. Unweighted Pair Group Method with Arithmetic Means (UPGMA) Cluster Analysis of Vaginal Microbiota in Each Group

UPGMA clustering was performed as a type of hierarchical clustering method. The evolution distance between samples can be observed by the distance of the branches and the distance of the clusters. The smaller distance means the smaller difference in species diversity between the two samples. The ZDD group and the CON group are clustered in the same cluster tree, so the community compositions of vaginal microbiota in the ZDD group and the CON group are similar. But the CCM group and the CON group are clustered in the different cluster tree, so the community compositions of vaginal microbiota in the CCM group are significantly different from the CON group (Figure 3(b)).

## 4. Discussion

Through our daily observation of tumor size in tumor-bearing mice, we found that the speed of tumor growth in the ZDD group was slower than the CCM group. And as it shown in Table 1, the tumor inhibitory rate (TIR %) of the ZDD group was 37.90% (>30%), which shows that ZDD has an antitumor effect. This result may be related to the pharmacological effects of Chinese medicine in ZDD. Based on pharmacological studies of Chinese medicine, each Chinese medicinal material in ZDD has antitumor effect [15, 18, 26–36]. Particularly, the main active ingredient of *Poria cocos*, pachymaran, could significantly inhibit the proliferation and induce the apoptosis of HeLa cells [37]. As we know, cancer can effect the deterioration of the body general condition manifested in dispiritedness, loss of appetite, and shorter lifetime. Our findings are in full agreement with it. Tumor-bearing mice had different degrees of pathological symptoms, such as dispiritedness, the pelage lost luster, the shapes of feces were changed, and food intake was reduced. But after ZDD treatment, these symptoms were obviously improved and the survival time of the tumor-bearing mice was significantly prolonged. These may be related to the recovery of the immune function of the body [38]. After ZDD treatment, with the improvement of systemic symptoms, the immune function of tumor-bearing mice was also restored, so the antitumor effect was reflected. This also suggests that Chinese medicine has certain advantages in improving the clinical symptoms, enhancing the quality of life, and prolonging the survival time of tumor patients. Therefore, it is worth developing Traditional Chinese Medicine as a major supplementary drug of treatment cancer.

Clinical studies confirm that the occurrence of cervical cancer is mainly caused by long-term chronic inflammation caused by HR-HPV infection. Chronic inflammation of female genital tract has often caused the disordered vaginal microbiota. Meanwhile, the disordered vaginal microbiota also increased the inflammation of the vagina and formed a vicious circle. As for the specific symptoms of cervical cancer, we observed the mice of the CCM group secreted unequal amounts of vaginal secretions, but this symptom was not observed in the CON and the ZDD groups. So, the occurrence of this symptom may be associated with the disordered vaginal microbiota. For further verification, we performed 16SrRNA gene sequencing analysis of the vaginal microbiota. Results showed that *Lactobacillus* in the CCM group was significantly lower than the CON group. This is consistent with Orfanelli's research [39]. As we know, *Lactobacillus* is the dominant genus in the normal vaginal microbiota which produces lactic acids that maintain an acidic environment to inhibit the growth and resist the invasion of pathogenic bacteria [21, 22]. It may prevent tumor genesis caused by the invasive pathogenic bacteria [23]. On the other hand, *Lactobacillus* also produces nitric oxide (NO) by stimulating macrophages and disrupting the energy metabolism of cancer cell [40]. At the same time, by secreting various metabolites, exopolysaccharides (EPSs), phosphorylated polysaccharides, and peptidoglycan secreted by *Lactobacillus* can inhibit the spread of malignant tumors [41]. In short, *Lactobacillus* plays an important role in the protection of female reproductive tract health. As a result, with the decreased *Lactobacillus*, a series of serious symptoms and signs appeared in the CCM group. The survival time of tumor-bearing mice in the CCM group was also obviously shortened. But *Lactobacillus* in the ZDD group was significantly increased than the CCM group, indicating that ZDD may have effect on the growth and reproduction of *Lactobacillus*.

Besides, our data showed that the relative abundance of *Pasteurella* (27.20%) and *Helicobacter* (18.49%) in the CCM group was much higher than the CON group (0.26%, 0.20%). Studies have shown that the number of *Pasteurella* often increases significantly when female reproductive tract inflammation occurs. It has been confirmed that *Pasteurella* is the main pathogen of female reproductive tract inflammation [42]. For another, *Helicobacter bilis* (*H. bilis*) is one of the main bacterial species of the CCM group. It is an enterohepatic *Helicobacter* species which may induce disease in susceptible animals [43]. *H. bilis* is known to be responsible for chronic liver damage and hepatobiliary cancer [44]. Moreover, *H. bilis* infection is associated with chronic intestinal inflammation [45] which shows its negative effect on the normal microbiota. Our data indicated that the presence of numerous pathogens caused the disordered vaginal microbiota of the CCM group. The changes in vaginal microbiota affect the occurrence and development of cervical cancer. So, we believed that *Pasteurella* and *Helicobacter* may play an important role in the occurrence and development of cervical cancer. This is in line with the recent analysis that has identified potential links between the microbiota and gynecological cancers [22]. However, the relative abundance of *Pasteurella* (0.80%) and *Helicobacter* (3.16%) in the ZDD group was much reduced compared to that in the CCM group. This suggests that ZDD has a certain inhibitory effect on *Pasteurella* and *Helicobacter*.

Furthermore, we have also found a phenomenon worthy of further study. In the particular acidic environment of the vagina, *Lactobacillus* is the dominant genus under normal circumstances which is the result of the CON group. With the occurrence of cervical cancer, a large number of pathogens occupy the dominant position; in our research, they are mainly *Pasteurella* and *Helicobacter*. Also in the case of cervical cancer, however, in the ZDD group, not only the dominant position of *Lactobacillus* recovered slightly, and the most special one is that, the emergence of the dominant position of *Staphylococcus* which is not observed in the other two groups. Among these, *Staphylococcus lentus* (*S. lentus*) is the main species of it. *S. lentus* is a coagulase-negative *Staphylococcus* (CNS) and belongs to normal microbiota which colonizes in the skin and mucous membranes [46]. Normally, this kind of microorganisms provides essential nutrients for their host and could prevent the invasion of pathogenic bacteria [47]. However, in recent years, the issue of *S. lentus* causing animal diseases has gradually attracted attention [48]. This may be related to the fact that the normal microbiota can be transformed into opportunistic pathogen under the condition of host immunity decrease, translocation, and dysbacteriosis of microbiota. Therefore, *S. lentus* still belongs to the less pathogenic bacteria. In addition, Faseela reported that the bioactive compounds produced by the *S. lentus* can inhibit the growth and reproduction of pathogenic bacteria [49]. According to our results and other studies, *S. lentus* is neither the dominant strain in normal vaginal microbiota nor the dominant strain in cervical cancer [21, 22], but it is the dominant strain in the ZDD group. This may suggest that ZDD has the effect of promoting the growth of *S. lentus* then to effectively inhibit pathogenic bacteria. Therefore, *S. lentus* may play an important role in regulating vaginal microbiota disorders. As a result, we thought that the ZDD anticervical cancer effect and the restoration of vaginal microbiota shown in this study are not only related to *Lactobacillus*, but also to *Staphylococcus*. It may be the result of the joint action of multiple groups, species, and strains of microbiota. Meanwhile, *S. lentus* may also provide new target bacteria for antitumor and anti-inflammatory effect. The specific mechanism of action among ZDD, *Staphylococcus*, and cervical cancer is worth exploring in the next step.

In the vaginal microbiota of mice treated with ZDD not only the amount of *Lactobacillus* and *Staphylococcus* increased, but also the amount of *Pasteurella* and *Helicobacter* reduced. At the same time, the relative abundance of the dominant taxa, in terms of microbiota structure, species abundance, and community compositions in the ZDD group, was more similar to the CON group. Therefore, we think that ZDD may have a regulatory effect in the disorder of vaginal microbiota. This regulatory effect is different from that of the antibiotics. It does not directly kill pathogens but increases the number of normal dominant bacteria and reduces the number of pathogens to regulate community compositions and others. Then, it turns the disordered vaginal microbiota to the normal microbiota state eventually.

The skin and the mucosal barrier are a major component of the mucous immune system. Mucosal local normal microbial flora and its metabolites play an important role in the production, differentiation, and proliferation of T cells. And the normal development and differentiation of T cells is necessary to maintain mucosal microbial homeostasis [50]. They play a vital role in resisting pathogen invasion and protecting the health of the body [51]. The disorder of the mucosa microbiota will result in a decrease in the mucosa local immune function and even systemic immune function. This may lead to the emergence of diseases and even the occurrence of tumors [22]. Our result is consistent with recent research. According to the pharmacological studies of Chinese medicine, *Cortex Phellodendri* and *Moutan Cortex* have the role of antipathogenic microorganisms [18, 29]. Through the establishment of normal vaginal microbiota, the local immune function of mucous membrane was restored. The aim of controlling vaginal inflammation and inhibiting the development of cervical cancer was achieved. So, we consider that the regulatory effect of ZDD on vaginal microbiota may be achieved by inhibiting the growth of pathogenic bacteria and promoting the growth of probiotics. Finally, by enhancing the mucosa, local and systemic immune function achieves the purpose of anticervical cancer. In recent years, tumor immunotherapy represented by CTA-4 and PD-1 has attracted wide attention. Some scholars have found that changes in the microbiota have an important effect on the efficacy of cancer immunotherapy [52]. This also indicates that changes in the local microbiota of the mucosa have an antitumor effect. Interestingly, the diversity of vaginal microbiota in ZDD group was greater than that in the CON group and the CCM group. This is not consistent with Gao's study [53]. But this may indicate that in the processes of ZDD stimulating the growth of probiotics, inhibiting the growth of pathogenic bacteria, and regulating the imbalance of the vaginal microbiota caused by cervical cancer, the vaginal microbiota community structure may rebuild. Next step, we will focus on the relationship between related bacteria and cervical cancer in order to know how they mediate the risk of human papillomavirus- (HPV-) induced cervical cancer. We believe that as a vital part of complementary and alternative medicine, it is very promising to exploit much more effective antitumor drugs from Chinese medicine.

In conclusion, this study reveals that the cervical cancer is associated with vaginal microbiota disorder. It is reported that ZDD may have a certain inhibitory effect on cervical cancer, and one of the pharmacological mechanisms of this action may be related to the restoration of normal vaginal microbiota. Further works are needed to explore the pharmaceutical mechanisms of ZDD. Although the relatively low number of mice was a limitation of the study, these findings enhance our understanding of the influence of changes in vaginal microbiota on the pathogenesis of cervical cancer and pharmacological mechanism of Chinese medicine. It also points out the direction for our further research work.

## 5. Conclusions

The inhibitory effect of ZDD on cervical cancer was revealed for the first time. One of the mechanisms of this effect may be achieved by the regulation of vaginal microbiota to achieve. Moreover, this function is not the regulation of single bacterial species and quantity, but multistrain, multilevel regulation and finally restores the balance of vaginal microbiota. Traditional Chinese herbal formulation can act on the multiple targets through their multiple components and play an integral role in the key biological process of disease occurrence and development, which promotes the body back to equilibrium, and thus, they play a therapeutic role.

In this study, we explored the effects of ZDD and vaginal microbiota on cervical cancer from a large perspective. Next step, based on our research data, researchers can further explore their relationship and mechanism deeply from specific antitumor or antipathogenic microbes' pharmaceutical composition and specific vaginal genus. We believe that as a vital part of complementary and alternative medicine, it is very promising to exploit much more effective antitumor drugs from Chinese medicine.

## Figures and Tables

**Table 1:** Tumor weight ($\bar{X}$ ± *S*), TIR%, survival time (*M* ± *Q*), and food intake ($\bar{X}$ ± *S*).

Group	*N*	Tumor weight (g)	TIR%	Survival time (days)	Food intake (g)
CON	12	NA	NA	21.00 ± 0.00	5.73 ± 0.50
CCM	12	1.64 ± 1.18	NA	19.50 ± 6.25^*∗*^	4.58 ± 1.10^*∗∗*^
ZDD	9	1.02 ± 0.69	37.90	21.00 ± 0.00	5.27 ± 0.74

*Note*. ^*∗*^*P*=0.011, ^*∗∗*^*P*=0.028. Tumor inhibitory rate (TIR) % = (1−Wt/Wn) × 100%, where Wn is the average tumor weight of the CCM group and Wt is the average tumor weight of the ZDD group. The survival time statistics are based on the 21 days of the experiment.

**Table 2:** Relative abundance of the top 10 dominant taxa at the species level (%).

Taxonomy	CON	CCM	ZDD
[Pasteurella]_pneumotropica	0.16	27.20	0.75
*Helicobacter*_bilis	0.01	10.81	0.03
Staphylococcus_lentus	0.01	0.01	12.03
Proteus_mirabilis	0.00	0.01	6.74
Pantoea_ananatis	1.48	0.71	2.13
Lactobacillus_johnsonii	1.53	0.02	0.48
Lactobacillus_reuteri	1.21	0.13	0.48
Lactobacillus_iners	0.00	0.00	2.33
Lactobacillus_agilis	0.69	0.12	0.65
Fusobacterium_mortiferum	0.63	0.10	0.13
Others	94.28	60.89	74.25

## Data Availability

Data supporting the results of this study can be obtained from the corresponding author upon request.
